# Rapid and Low-Cost Detection and Quantification of SARS-CoV-2 Antibody Titers of ICU Patients with Respiratory Deterioration Using a Handheld Thermo-Photonic Device

**DOI:** 10.3390/biomedicines10061424

**Published:** 2022-06-15

**Authors:** Damber Thapa, Nakisa Samadi, Andrew Baker, Claudia dos Santos, Uriel Trahtemberg, Nima Tabatabaei

**Affiliations:** 1 Department of Mechanical Engineering, York University, Toronto, ON M3J 1P3, Canada; dthapa@yorku.ca (D.T.); nakisasa@yorku.ca (N.S.); 2 Interdepartmental Division of Critical Care, St. Michael’s Hospital, Toronto, ON M5B 1W8, Canada; andrew.baker@unityhealth.to (A.B.); claudia.dossantos@unityhealth.to (C.d.S.); 3 Critical Care Department, Galilee Medical Center, Nahariya 2210001, Israel; uriel.trahtemberg@unityhealth.to; 4 Keenan Research Centre of Biomedical Science, St. Michael’s Hospital, Toronto, ON M5B 1W8, Canada

**Keywords:** COVID-19, coronavirus, antibodies, lateral flow immunoassay, photothermal, SARS-CoV-2

## Abstract

While research suggests that COVID-19 vaccines are effective in producing anti-SARS-CoV-2 antibodies that reduce the risk of COVID-19 and its potentially severe complications, how long these antibodies persist after the infection/vaccination is unknown. Longitudinal studies and rapid and scalable platforms are needed for large-scale sero-diagnosis and vaccine evaluation. In this study, we examine the efficacy of our recently-developed handheld thermo-photonic device for rapid and low-cost assessment of the adaptive immune response of COVID^+^ and COVID^−^ patients admitted to the intensive care unit (ICU) at a local hospital due to respiratory deterioration. Antibody testing included detection and quantification of IgG and IgM via thermo-photonic sensing of a commercially available COVID-19 IgG/IgM rapid test as well as standard measurements with quantitative enzyme-linked immunosorbent assays (qELISA). The results demonstrate that the thermo-photonic reader in conjunction with COVID-19 IgG/IgM test cassettes can detect and quantify IgG levels in COVID-19 antibody assays within the clinically relevant range and with a high correlation to those obtained from qELISA. We also found that the IgG antibody is more reliable for detecting individuals with an adaptive immune response to SARS-CoV-2 compared to the IgM antibody. The developed reader offers a low-cost, portable, and scalable solution for accessing the antibody titer of individuals against SARS-CoV-2 and can be used in local hospital settings.

## 1. Introduction

The humoral immune response is initiated when an individual is infected with severe acute respiratory syndrome coronavirus 2 (SARS-CoV-2) to protect them against the infection [1]. An analogous pattern of immune response occurs after the administration of vaccines [2,3]. Such responses are believed to be key to immunity against the COVID-19 virus, even for individuals previously infected with SARS-CoV-2 [4]. Consequently, public health units around the globe strongly urge individuals to get vaccinated and to strive to maintain a high level of immunity through the administration of booster shot vaccines.

With widespread and global vaccinations, measurement of COVID-19 antibodies is emerging as a post-vaccination endemic need. For example, there is currently not enough evidence to know how long immunity induced by vaccination persists; longitudinal sero-epidemiological investigations are needed to know how well the vaccines work in the long run, especially with the ongoing emergence of highly transmissible new coronavirus variants with mutations in the spike protein and/or recombination of variants. Another example is recent studies showing that monitoring changes in the components of the immune response, such as anti-SARS-CoV IgG (Immunoglobulin G) in serum, is insightful for the evaluation of patient response to treatments [5] or effectiveness of the vaccines [6]. Assessing and monitoring the immune response at the individual, and also population, levels can become key to the success of our efforts for navigating through the outbreak.

Screening of humoral immune response is normally carried out through serological tests. Neutralization assays for measuring antibody titers remain the gold standard for examining antibody response against the virus; however, these laborious tests require live virus, highly skilled operators, and biosafety level 3 facilities [7]. Therefore, other laboratory tests, such as chemiluminescent assay (CLIA) and enzyme-linked immunosorbent assay (ELISA) are more common for serological diagnosis [8]. Although CLIA and ELISA do not require the use of a live virus, they are still expensive, time-consuming, and require laboratory facilities and trained personnel. These attributes make CLIA and ELISA not suitable for large-scale sero-diagnosis and vaccine evaluation studies. Large scale sero-epidemiological endeavors require simple, low-cost, and rapid immuno-chromatographic methods for not only detection, but also quantification of COVID-19 antibodies.

A rapid diagnostic test (RDT) is a fast, low-cost, and point-of-care approach for the detection of analytes in fluidic specimens. While COVID-19 antibody RDTs are widely commercialized, these convenient and scalable solutions provide essentially binary information about the presence of antibodies and cannot quantify the antibody level of infected and/or vaccinated individuals. The diagnostic performance of COVID-19 serological rapid tests was examined from the early days of the pandemic [9,10,11], leading to the consensus that these tests are not of significant value for detection of infection as they become reliable ~2–3 weeks post infection [11,12]. Owing to such response, COVID-19 antibody rapid tests were initially deemed of no significant value to the management of the crisis. Recently, however, several attempts have been made to quantify the antibody levels with COVID-19 antibody RDTs by means of rapid test reader instruments. In an approach, reflection of laser light from the RDT nanoparticles was used as a signal for the quantification of antibodies [13]. Spectral signatures of reflected light from RDTs [14,15] or surface-enhanced Raman scattering (SERS) [16] have also been used to enable the quantification of IgG/IgM antibodies with RDTs. Other recent attempts include magnetic transduction in RDTs that utilize magnetic nanoparticles as tag particles [17] and fluorescent expression measurement in RTDs incorporating fluorescence labeling dyes [18]. While the limit of detection and quantification performance of these readers are promising, the photon-counting [13] and spectroscopic [14,15,16] methods are benchtop systems containing bulky expensive equipment which limits their adoption for large scale studies. Methods utilizing magnetic [17] or fluorescent [18] tag particles are also limited by the cost of RDT and reader instruments making them not suitable for scalable administration at the points of need.

In the past few years, we have developed thermo-photonic sensing methodologies for the detection and quantification of target analytes using commercially available lateral flow immunoassay (LFA) RDTs [19,20,21]. These sensing innovations are based on the detection of infrared thermal emissions that are produced by RDT nanoparticles once excited with a laser. The major limitation of earlier works was that these solutions were either costly (>USD $8000) or bulky. In a recent work, however, we presented a low-cost and portable variant of our thermo-photonic sensing platform based on smartphone infrared camera sensors [22]. To further reduce the cost and size of the system, a handheld and portable device was also developed and tested on detecting and quantifying COVID-19 IgG antibody concentrations in control positive solution (also known as simulated solution) samples [23].

The current manuscript is focused on demonstration of the feasibility of detecting and quantifying COVID-19 antibody concentrations in clinical samples from patients admitted to the intensive care unit (ICU) with our low-cost and portable thermo-photonic device. Results from the thermo-photonic reader device are compared to those of laboratory-based quantitative ELISA measurements. Standard RT-PCR testing is used for confirmation of COVID-19 infection. Results show that the portable thermo-photonic device in conjunction with commercially available and low-cost colloidal nanoparticle-based assays can detect and quantify antibody levels in human serum samples within the clinically relevant range (especially for IgG antibodies). As such, this work is expected to open the door for rapid, low-cost, portable, and scalable measurement of COVID-19 antibodies which can be key to the success of the sero-epidemiological endeavors in the endemic era.

## 2. Materials and Methods

### 2.1. Ethical Statement 

The study was conducted under the Declaration of Helsinki and it was approved by the Research Ethics Boards of St. Michael’s Hospital (20-078—COLOBILI) and York University (e2020-250). The clinical trial is registered with United States National Library of Medicine under name COVID-19 Longitudinal Biomarkers in Lung Injury (COLOBILI; NCT04747782). Informed consent was obtained from all patients or their legal surrogates. 

### 2.2. Samples and Processing 

All human serum samples were collected for the “COVID19 Longitudinal Biomarkers in Lung Injury” (COLOBILI) study at St. Michael’s Hospital in Toronto, Canada. All patients were critically ill with respiratory disease in the intensive care unit (ICU). Five COVID^+^ and four COVID^−^ patients were selected for longitudinal sampling. Twenty-eight (N = 28) longitudinal blood samples were collected from COVID-19 positive (COVID^+^) patients; sixteen (N = 16) longitudinal blood samples were collected from COVID-19 negative (COVID^-^) patients. COVID status was determined for all patients upon admission to the ICU unit (i.e., day 0) using polymerase chain reaction (PCR) of nasopharyngeal swabs and/or endotracheal aspirates as per hospital laboratory medicine protocol. The average age of the COVID^+^ and COVID^−^ patients was 73 (range 61–83 years) and 74 (range 63–92 years), respectively. After blood was drawn from a patient, it was left to coagulate on ice for 30 min, followed by centrifugation at 1300× *g* for 10 min. The serum was then separated and frozen at −20 °C on-site, and then moved to −80 °C for storage within 48 h. They were thawed and refrozen only once for aliquoting before the time of antibody testing.

### 2.3. COVID-19 IgG/IgM Assays 

Commercially available colloidal gold nanoparticles (GNPs) based immunochromatographic test cassettes (BTNX Inc., Rapid Response^TM^ COVID-19 IgG/IgM Rapid Test Device, Markham Ontario) were used in this study. These kits were designed with the nucleocapsid and spike receptor-binding domain (RBD) antigens to detect SARS-CoV-2 IgG and IgM antibodies in the whole blood, plasma, or serum samples. These assays were spiked according to the manufacturer’s guidelines [24]. Briefly, 5 µL of the serum sample was dispensed in the sample well, followed by the addition of two drops of assay buffer. The sample flows along the strip due to the capillary action and the target antibodies (IgG/IgM) if present, bind with the antigen-GNP conjugates making an antigen antibodies complex. This complex further migrates and eventually accumulates on the test lines to create visual contrasts. Similar visual contrast is produced in the control line due to the accumulation of antigen-GNP conjugates. Therefore, a SARS-CoV-2 IgG^+^ test shows a colored line at the IgG line in the cassettes, a SARS-CoV-2 IgM^+^ test shows a colored line at the IgM line, and the appearance of two-colored lines at IgG and IgM in the test cassettes indicates the presence of both IgG and IgM anti-SARS-CoV-2 antibodies, whereas the presence of a colored line at the control line suggests a properly spiked test. In such immunodiagnostic assays, the color intensity of the test lines correlates with the concentration of the IgG/IgM in the serum.

### 2.4. Thermo-Photonic Interpretation of IgG/IgM Assays 

**Figure 1:**
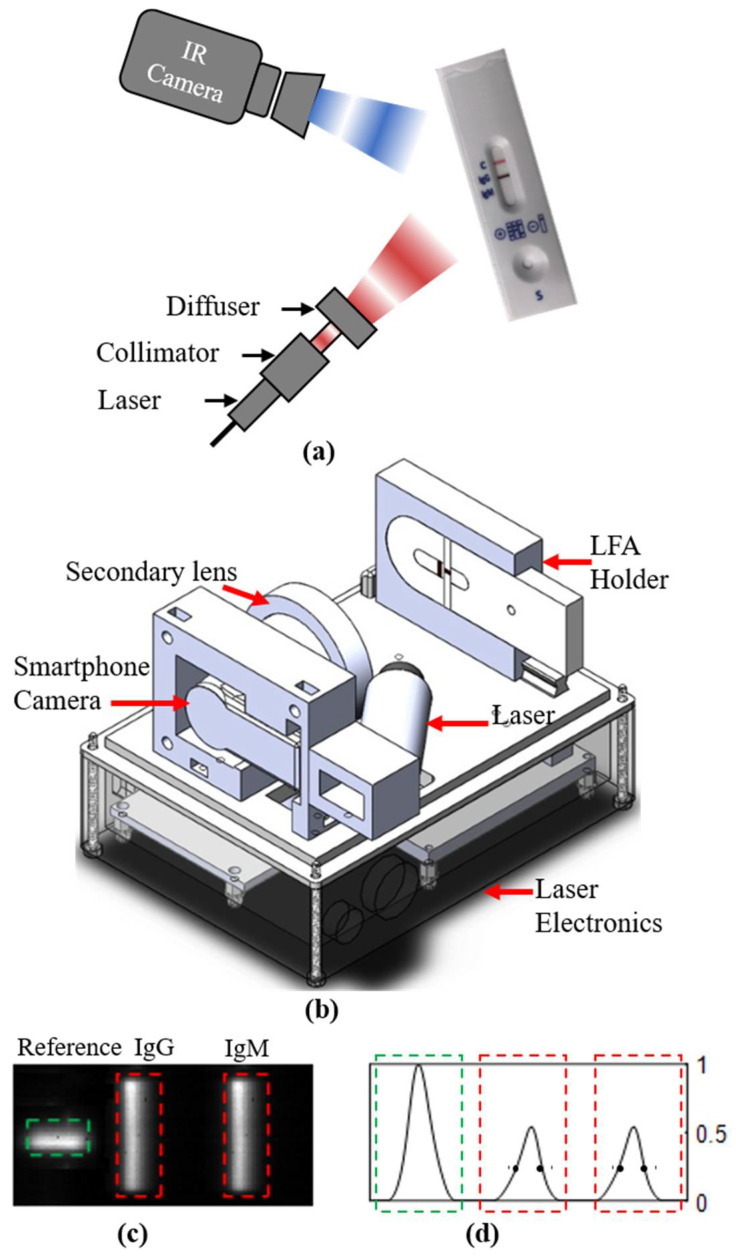
Schematic showing (**a**) the working principle and (**b**) exploded view of handheld thermo-photonic device used for interpretation of assays. (**c**) Lock-in thermography (LIT) amplitude image of an assay. The green rectangle shows the reference IgG and red rectangles show the test IgG and IgM bands. (**d**) Three gaussian-shaped curves produced by averaging pixels along the longer axis direction of reference (green rectangle) and IgG and IgM bands (red rectangles). Amplitude metric was calculated by averaging the intensity between two solid dots on each IgG and IgM curves.

A handheld thermo-photonic device that operates on the principle of Lock-in-Thermography (LIT) is used in this study. The exploded view of the LIT device is shown in Figure 1b. While detailed information about the system can be found elsewhere [23], in brief, a low-cost 808 nm laser diode installed in a cylindrical laser-lens housing (Besram Technology Inc., Wuhan, China) was used as an excitation source. A laser driver (Wuhan Jingluyao Trading Co., Wuhan, Hubei, China) was used to provide modulated electric current to the laser driver created by a 1 Hz TTL signal received from a generic PWM circuit. The laser provides 600 mW of optical power in a rectangular format that is enough to cover the IgG, IgM, and control lines of the assay. The electric power on the laser diode was supplied by a generic timer relay circuit for 30 s upon push button triggering. Upon heating, a low-cost smartphone infrared camera (Seek Thermal Compact Android) in conjunction with a secondary low-cost Zinc Selenide lens (MCW laser Inc., Wuhan, China) was used to detect the infrared radiation emitted from the assay. All the laser electronics and device hardware were held inside a 3D printed enclosure (Figure 1b), and insertion of the COVID-19 antibody test cassette was accomplished through a sliding mechanism. The overall size and cost of the handheld device are 8 × 11 × 10 cm^3^ and ∼US $350, respectively. 

A custom-made software development kit (SDK), which was developed in C# and LabVIEW, was used for the acquisition of infrared frames through the USB 2.0 interface [22]. An amplitude image of the test strips was obtained via lock-in demodulation of acquired infrared thermal frames in the LabVIEW environment (Figure 1c). Unlike test lines which immobilize the colloidal GNPs, the control line of the assay used in this study immobilizes latex (tag particles); therefore, the light absorbed by the control line is negligible. Thus, a test line of an assay spiked with 10 µg/mL of IgG was cut and permanently placed in the field of view of the device and always imaged together with the test assay. The signal from the reference line was used for the signal normalization that minimizes the systematic errors induced by day-to-day variations in laser illumination and improves the repeatability of the device. The amplitude image was post-processed for calculating the amount of heat (also known as thermal wave amplitude) captured by the nanoparticles immobilized on the test line. In the post-processing, the amplitude image was normalized, followed by averaging the intensity of pixels over all the rows in the strip to generate three Gaussian-shaped carvers at IgG, IgM, and control line positions (Figure 1d). Amplitude metrics which are obtained by taking average intensities within full width at half maximum (FWHM; between two solid circles in Figure 1d) [23] of the IgG and IgM Gaussian curves were calculated for the quantitative comparison of IgG/IgM in assays spiked with different serum samples of COVID^+^ and COVID^−^ patients. All thermo-photonic measurements were repeated three times and the data were reported as the mean of repeated measurements with standard deviation error bar.

### 2.5. ELISA

To evaluate the detection and quantification performance of the handheld thermo-photonic device, antibody titers of all clinical samples were measured with quantitative enzyme-linked immunosorbent assays (ELISA) as a reference. ELISA tests (ImmunoDiagnostics Limited, Hong Kong) were performed for the measurement of IgG and IgM antibodies against S1 protein of the SARS-CoV-2 virus in the serum samples collected from the patients. Two separate quantitative ELISA kits were used for detection and quantification of IgG and IgM antibodies. Both kits were two-step incubation immunoassay kits. The kits included 96-well plates, coated with SARS-CoV-2 S1 protein that captures IgG and IgM antibodies against SARS-CoV-2 S1 protein in the sample. After washing away the unbound materials, captured IgG and IgM against SARS-CoV-2 S1 protein was detected by anti-human IgG and IgM polyclonal antibodies conjugated with horse radish peroxidase (HRP). After the washing step, the chromogenic substrate 3,3′,5,5′- tetramethylbenzidine (TMB) was added, resulting in generation of color density proportional to the amount of anti- SARSCoV-2 S1 antibodies captured inside the wells. The color density of wells was measured with a standard 450 nm plate reader and converted into ng/mL using calibration curves. Calibration curves and limit of detection were determined experimentally and found to be 0.625 ng/mL for IgG antibodies, and 0.031 ng/mL for IgM antibodies. All ELISA tests were performed in duplication according to the manufacturer’s instructions; the data were reported as the mean of duplicate measurements with standard deviation error bar. The analyses were performed at the Analytical Facility for Bioactive Molecules, The Hospital for Sick Children, Toronto, ON, Canada.

### 2.6. Statisical Analysis 

The *t*-test was performed to find the statistical difference in mean amplitude metrics between two different groups following the normality test. A regression analysis was performed to find the relationship between ELISA and thermo-photonic tests. The receiver operating characteristic (ROC) curves were plotted to examine the performance of the thermo-photonic system for detecting antibody^+^ and antibody^−^ sera. The statistical analysis and plotting were carried out with R (Version 4.12). *p* < 0.05 was considered statistically significant. 

## 3. Results and Discussion 

To examine the performance of the developed thermo-photonic device, titers of IgG and IgM antibodies in clinical samples from COVID+ and COVID− patients were determined using both quantitative ELISA and the developed handheld thermo-photonic device. Results were then compared statistically to examine the feasibility of assessing the human adaptive immune response to COVID virus with the developed technology in a rapid and low-cost manner.

As outlined in Section 2, thermo-photonic measurements were based on commercially-available immune-chromatographic test cassettes. Upon spiking of cassettes, antigen-GNP-analyte complexes are accumulated at the test lines. Such accumulations result in creation of colored lines due to the reflection of ambient light from the surface of GNPs immobilized on the test line. A higher concentration of analyte immobilizes a larger number of GNPs, creating a stronger colorimetric signal on the test line. However, the immobilized GNPs also absorb certain wavelengths of light (e.g., 808 nm) very efficiently and convert the absorbed light into heat due to the surface plasmon resonance [25]. Taking advantage of this physical phenomenon, our portable and low-cost device illuminates the RDT with laser excitation of 808 nm while monitoring the infrared thermal response with a cellphone attachment infrared camera, Figure 1a. To decouple the effect of ambient temperature from measurements, the laser is intensity-modulated at a frequency of 1 Hz and RDT infrared responses are correspondingly demodulated at 1 Hz (also known as lock-in-thermography or LIT). In such a sensing arrangement, the intensity-modulated laser excitation is selectively absorbed by the GNPs on the assay and produces an amplified thermal wave compared to the non-absorbing surrounding nitrocellulose paper. The selective absorption of light further reduces the baseline/noise signals from the surrounding medium, which increases the sensitivity of the device, leading to the successful detection of photo-thermal signals from even a small number of GNPs. Since the concentration of the target analyte (e.g., IgG or IgM) correlates with the number of GNPs immobilized at the test lines, the amplitude of the measured signals at test lines can be used to quantify the analyte concentrations [23].

### 3.1. Evaluation of IgG Titer 

**Figure 2:**
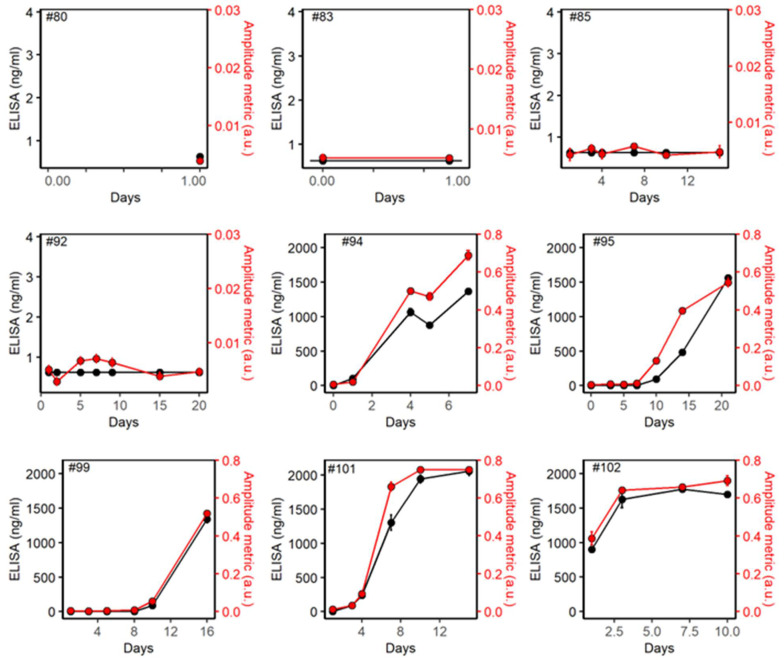
Plots for IgG antibodies. ELISA (y1 axis) and IgG amplitude metrics (y2 axis) with respect to days of ICU for COVID− (IDs #80, 83, 85 and 92) and COVID+ (IDs #94, 95, 99, 101 and 102) participants. Individual data points show mean ± standard deviation of 2 measurements for ELISA and 3 measurements for thermo-photonic methods.

Figure 2 shows the variation of IgG antibodies with respect to the days of ICU admission for COVID^−^ patients (patient IDs 80, 83, 85, and 92). The y1 axis represents the standard ELISA test and the y2 axis represents the amplitude metric from the thermo-photonic device. Both ELISA and thermo-photonic systems show negative results for COVID^−^ patients. Figure 2 also shows the same variations for the COVID^+^ patients (Patient IDs 94, 95, 99, 101, and 102). These plots demonstrate that longitudinal IgG measurements from ELISA and thermo-photonic device are highly correlated. Table 1 summarizes the results of PCR, ELISA, and thermo-photonic systems for all COVID^−^ and COVID^+^ samples.

Table 1 shows that IgG antibodies failed to develop detectable levels (ELISA > 0.625 ng/mL) in all of the COVID^+^ patient’s samples on the first day of ICU admission. The results are in line with those previous reports which show antibodies specific to SARS-CoV-2 are detectable in blood 1–3 weeks after initial infection and then increase rapidly [11]. Although we do not know the exact day the patients were infected by the SARS-CoV-2 virus, they all had respiratory deterioration due to COVID-19 and were admitted to ICU.

Data in Table 1 suggests that IgG antibodies were detected in all patients after a few days of ICU admission. Therefore, total serum samples were further divided into antibody^+^ and antibody^−^ samples based on the ELISA readings. ELISA reading >0.625 ng/mL were considered antibody^+^ and <0.625 ng/mL were considered antibody^−^ specimens as an ELISA reading of 0.625 ng/mL was the threshold for IgG. Of the COVID^+^ samples, 18/28 developed SARS-CoV-2 specific IgG antibodies. All COVID^−^ patients showed negative results for the ELISA test (<0.625 ng/mL).

**Figure 3:**
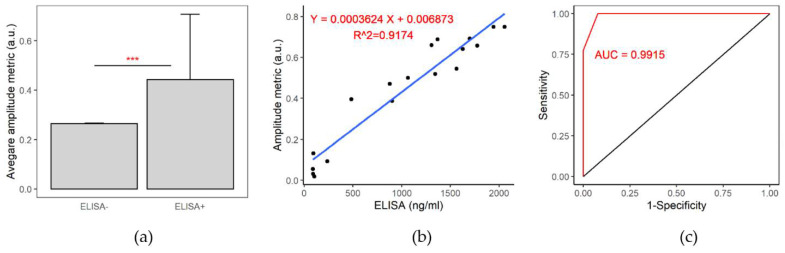
(**a**) A bar diagram showing average IgG amplitude metrics between the ELISA− and ELISA+ samples. The average IgG amplitude metrices is significantly higher in ELISA+ samples (*** indicates *p* ≤ 0.001). (**b**) A linear regression plot between the IgG amplitude metric and ELISA for IgG. (**c**) A ROC curve that shows the performance of our device for detecting ELISA− and ELISA+ IgG samples.

Mean amplitude metrics of the antibody^+^ and antibody^−^ samples were calculated and compared (Figure 3a). The mean amplitude metric of antibody^+^ samples was significantly higher than that of the antibody^−^ samples (*t*-test, *p* < 0.001). A scatter plot (Figure 3b) depicts the relationship between the IgG amplitude metric and IgG antibody titer calculated by ELISA. The Pearson’s correlation coefficient (*p* = 0.96) indicated that the amplitude metric is highly correlated with ELISA. The cut-off value of our thermo-photonic device was determined by selecting the highest proportion of correctly classified IgG^+^ and IgG^−^ samples. The thermo-photonic device was able to discriminate between IgG^+^ and IgG^−^ samples at an amplitude metric value of 0.015 (a. u.) with 100% sensitivity and 100% specificity (note sample size was limited to N = 44). Hence, the sample was considered IgG^+^ for the thermo-photonic device if the amplitude metric was greater than the cut-off value. A best-fit line (regression line) was added to the plot to find an association between two variables (Figure 3b). The high value of R-squared (R2 = 0.92) indicates that all the data points fall around the fitted regression line. The model can be used as a calibration line in the future to predict the concentration of IgG in serum using the thermo-photonic device.

To determine the sensitivity of the thermo-photonic system to differentiate IgG+ and IgG− samples, a receiver operating characteristic (ROC) curve was drawn (Figure 3c). True positive and true negative samples were determined based on the IgG^+^ and IgG^−^ by ELISA. A high value of area under the ROC curve (AUC = 0.99) indicates the high predictive ability of the thermo-photonic system for distinguishing IgG^+^ and IgG^−^ sera. These analyses demonstrated that thermo-photonic device provides similar IgG titer as those provided by quantitative ELISA, albeit at a much lower cost and at the point of care. 

### 3.2. Evaulation of IgM Titer 

**Figure 4:**
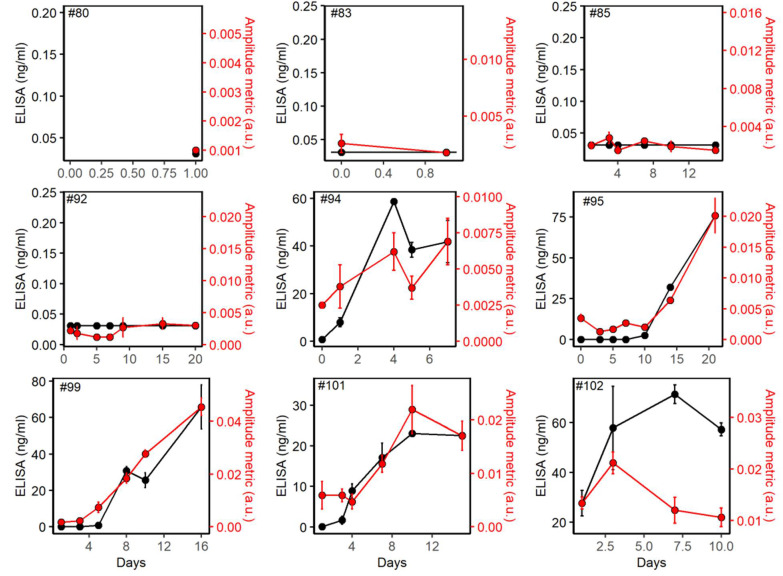
Plots for IgM antibodies. ELISA (y1 axis) and IgM amplitude metrics (y2 axis) with respect to days of ICU for COVID− (IDs #80, 83, 85 and 92) and COVID+ (IDs #94, 95, 99, 101 and 102) participants. Individual data points show mean ± standard deviation of 2 measurements for ELISA and 3 measurements for thermo-photonic methods.

IgM antibodies were also analyzed in a way similar to IgG. Figure 4 shows the variation of IgG antibodies with respect to the days of ICU admission for COVID^−^ patients (patient IDs 80, 83, 85, and 92) and COVID^+^ patients (Patient IDs 94, 95, 99, 101, and 102). Y1 axis (left) shows the ELISA evaluations while the y2 axis (right) shows the thermo-photonic evolutions. IgM levels of COVID^+^ patients were considerably higher than those of the COVID^−^ control group. ELISA reading >0.031 ng/mL was considered as IgM^+^ and <0.031 ng/mL was considered as IgM^−^ specimens. We observed high prevalence and titers of IgM among the COVID+ patients; 21/28 (75%) of the sample had SARS-CoV-2 specific IgM antibodies. Three serum samples that were IgG negative, were found to be IgM positive. As suggested by other studies, this may reflect earlier appearance as well as the disappearance of IgM compared to IgG in the course of COVID [26].

**Figure 5:**
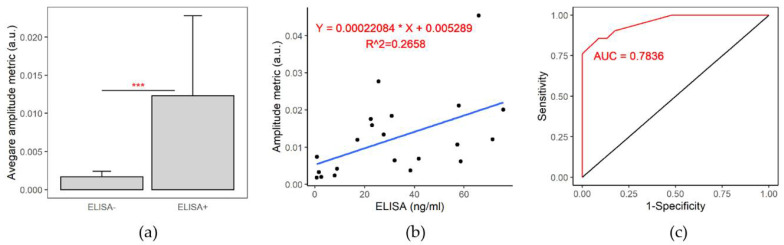
(**a**) A bar diagram showing average IgM amplitude metrics between the ELISA− and ELISA+ samples. The average IgM amplitude matrices of the ELISA+ samples is significantly higher than that if ELISA- samples (*** indicates *p* ≤ 0.001). (**b**) A linear regression plot between the IgM amplitude metric and ELISA for IgM. (**c**) A ROC curve that shows the performance of our device for detecting ELISA− and ELISA + IgM samples.

Mean amplitude metrics between IgM^+^ and IgM^−^ samples were calculated and compared (Figure 5a). Although the mean amplitude metric of IgM^+^ samples was smaller than that of IgG, it was significantly different with IgM^−^ samples (*t*-test, *p* < 0.001). Figure 5b shows the scatter plot between the amplitude metric at the IgM line and ELISA IgM^+^ readings. Unlike IgG, IgM values are spread further away and the correlation between IgM and IgM amplitude metric is low (correlation coefficient = 0.52). The reader was able to discriminate between IgM^+^ and IgM^−^ samples at an IgM amplitude metric value of 0.0041 (a. u.) with 90% sensitivity and 100% specificity. The relationship between ELISA IgM is further accessed with the IgM amplitude metric by adding the best fit line (regression line) in the scatter plot (Figure 5b). The observed IgM values are more dispersed from the fitted regression line with an R^2^ = 0.26 value much lower than that of IgG. The prediction of IgM by our thermo-photonic system appears to be less reliable compared to IgG. The lower sensitivity of the thermo-photonic system for classifying IgM^+^ and IgM^−^ sera is also shown by the lower value of AUC (=0.78) in the ROC curve (Figure 5c). The reason behind this discrepancy is two-fold. Firstly, the nominal limit of detection of the rapid test used in our thermo-photonic measurements is much inferior for IgM (210 ng/mL) compared to IgG (3.4 ng/mL) which, in return, makes the rapid tests less sensitive at low IgM titers. Secondly, the clinical samples used in our study had very low titers of IgM because the donors were beyond the initial days of viral infection. Studies have shown that IgM titers rapidly decline after the initial days of infection due to seroconversion and class switching, leading to elevation of IgG titers, and thus, long-term immunity [27]. Consistent with such studies, our ELISA measurements show significantly larger titers of IgG than IgM in the clinical sera (Figure 2 vs. Figure 4). Nevertheless, the developed rapid and low-cost device could detect presence of IgM antibodies at concentrations below the nominal LOD of rapid test (Figure 5a) due to the enhanced detection sensitivity and specificity of the LIT detection method. 

## 4. Conclusions

In this study, we examined the feasibility of detecting and quantifying COVID-19 IgG and IgM antibodies with a rapid, low-cost, and portable thermo-photonic device. Longitudinal blood samples (N = 44) from COVID^+^ and COVID^−^ patients admitted to the ICU unit were analyzed with the low-cost device and results were statistically compared to those obtained from quantitative ELISA. It was found that the thermo-photonic system is as sensitive as ELISA for the detection and quantification of IgG antibodies. At the cut-off amplitude metric value of 0.015, the anti-SARS-CoV-2 IgG sera can be identified from the control at 100% sensitivity and specificity. We also observed that, compared to IgM, IgG levels were significantly higher in the COVID^+^ sera; IgG, therefore, can be a better metric for identifying individuals with an adaptive immune response to SARS-CoV-2. These results are in agreement with other recent reports that IgG is perhaps a stronger indicator for immunity to COVID-19 infection [28,29]. The cross-reactivity and substance interference showed that the performance of the IgG/IgM assay used in this study is not affected by antibodies of common human diseases or other substances normally present in the human serum/blood [24]. The thermo-photonic device in conjunction with IgG/IgM antibody cassettes provides a reliable method for the serodiagnosis of infected and/or vaccinated individuals and is suitable for testing the immune response of patients in local hospital settings. It should be noted that the antibody test does not detect viruses; therefore, the device should be used for detecting and quantifying antibodies specific to SARS-CoV-2, but not for any other viruses or pathogens. The participants in our cohort were enrolled when they were admitted to ICU with respiratory failure and suspected COVID, and the analysis on the dates of symptoms onset was not available. Nonetheless, both the ELISA and the thermo-photonic system failed to detect the antibody in the sera on admission day for some of the patients. The main limitation of this study is the limited sample size (N = 44); nevertheless, the sample size was deemed adequate for performing essential statistical analysis to demonstrate proof-of-concept and feasibility of longitudinal monitoring antibody levels of acutely ill COVID-19 patients at a low cost. Obtained results from the limited number of clinical samples appear to be in line with those published on larger COVID-19 cohorts [28].

## Figures and Tables

**Table 1:** Demographic information of patients and test results with the ELISA and thermo-photonic reader. Sign + refers to a positive test and − sign refers to a negative test.

ID	Age	PCR	ICU Days	ELISA		Our Reader	
				IgG	IgM	IgG	IgM
80	74	−	1	−	−	−	−
83	70	−	0,1	−	−	−	−
85	92	−	1,3,4,7,10,15	−	−	−	−
92	63	−	1,2,5,7,9,15,20	−	−	−	−
94	67	+	0	−	+	−	+
			1,4,5,7	+	+	+	+
95	78	+	0,3,5,7	−	−	−	−
			10,14,21	+	+	+	+
99	77	+	1,3	−	−	−	−
			5,8	−	+	−	+
			10,16	+	+	+	+
101	61	+	1	−	−	−	−
			3,4,7,10,15	+	+	+	+
102	83	+	1,3,7,10	+	+	+	+

## Data Availability

The data that support the findings of this study are available from the corresponding author, upon reasonable request.
